# Sensory Drivers of Consumer Acceptance and Behavioural Responses to Baobab Fruit Pulp (*Adansonia digitata* L.): Evidence from a Portuguese Convenience Sample

**DOI:** 10.3390/foods15152629

**Published:** 2026-07-27

**Authors:** Leandro Oliveira, Augusta Tomás, Vera M. S. Isca, Rebeca André, Raquel P. F. Guiné, António Raposo, Patrícia Rijo

**Affiliations:** 1 CBIOS (Research Center for Biosciences and Health Technologies), ECTS (School of Health Sciences and Technologies), Lusófona University, Campo Grande 376, 1749-024 Lisboa, Portugal; vera.isca@ulusofona.pt (V.M.S.I.); rebeca.andre@ulusofona.pt (R.A.); antonio.raposo@ulusofona.pt (A.R.); patricia.rijo@ulusofona.pt (P.R.); 2 ECTS (School of Health Sciences and Technologies), Lusófona University, Campo Grande 376, 1749-024 Lisboa, Portugal; 3 Research Institute for Medicines, iMed.ULisboa, Faculty of Pharmacy, Universidade de Lisboa, Av. Prof. Gama Pinto, 1649-003 Lisboa, Portugal; 4 Centro de Química Estrutural, Institute of Molecular Sciences, Faculdade de Ciências, Universidade de Lisboa, Campo Grande, 1749-016 Lisboa, Portugal; 5 CERNAS-IPV Research Centre for Natural Resources, Environment and Society, Polytechnic University of Viseu, Repeses, 3504-510 Viseu, Portugal

**Keywords:** Baobab fruit pulp, sensory analysis, consumer behaviour, hedonic perception, cluster analysis, food innovation

## Abstract

Baobab fruit (*Adansonia digitata* L.) has attracted increasing attention due to its nutritional value and potential as a functional food ingredient; however, its acceptance among European consumers remains poorly understood. This study investigated sensory drivers of liking, consumer segmentation, and behavioural responses towards baobab fruit pulp in a Portuguese convenience sample of adults. Sensory attributes were evaluated using a 9-point hedonic scale, alongside measures of intended consumption frequency and willingness to pay. Overall liking indicated moderate acceptance (median = 6; IQR: 3–7). Multiple linear regression identified taste as the main driver of liking (β = 0.362, *p* < 0.001), followed by texture (β = 0.182, *p* = 0.042) and acidity (β = 0.105, *p* = 0.045), explaining 82.3% of the variance in overall liking. Cluster analysis revealed two consumer segments: low-liking consumers (*n* = 70; median = 4) and high-liking consumers (*n* = 47; median = 8). High-liking consumers reported significantly greater intended consumption frequency and willingness to pay (*p* < 0.01). These findings highlight the importance of sensory optimisation and consumer heterogeneity in supporting consumer acceptance of baobab fruit pulp and informing future product development in European markets.

## 1. Introduction

The valorisation of underutilised plant-based foods has gained increasing attention in recent years, driven by the need to promote sustainable diets, diversify food systems, and enhance nutritional quality [1,2]. Among these resources, baobab fruit (*Adansonia digitata* L.), commonly known as múcua, stands out due to its remarkable nutritional profile, including high levels of vitamin C, dietary fibre, polyphenols, and organic acids with recognised antioxidant potential [3,4,5,6]. These characteristics have positioned baobab as a promising functional ingredient within the context of health-oriented food innovation [7,8].

Traditionally consumed in sub-Saharan Africa, baobab pulp plays an important role in local diets, being incorporated into beverages, porridges, and fermented products [9,10]. Beyond its nutritional relevance, baobab contributes to rural livelihoods and local economies, reinforcing its socio-economic importance [10,11]. The approval of baobab as a novel food ingredient in the European Union has facilitated its entry into new markets, promoting its use in functional and plant-based formulations [12]. However, the successful integration of such traditional products into Western food systems depends largely on consumer acceptance, which remains insufficiently explored [13].

From a sensory perspective, baobab pulp exhibits a distinctive profile characterised by pronounced acidity, mild sweetness, and a dry or powdery mouthfeel [3,7]. While these attributes are culturally familiar and appreciated in traditional contexts, they may represent a barrier to acceptance in populations with different sensory expectations. Consumer research consistently demonstrates that sensory properties, particularly taste and texture, are primary determinants of food liking and choice [14,15]. In the context of novel foods, unfamiliar sensory characteristics may reduce acceptance unless compensated by perceived benefits or repeated exposure [16].

In addition, consumer responses to novel food products are not homogeneous. Segmentation approaches have been widely used in sensory and consumer science to identify distinct preference patterns and to better understand heterogeneity in acceptance [13,17]. Such approaches are particularly relevant when evaluating products with unconventional sensory profiles, as they allow the identification of both more receptive and more critical consumer groups [18].

Beyond sensory evaluation, behavioural outcomes such as intended consumption frequency and willingness to pay are increasingly recognised as key indicators of consumers’ behavioural responses towards novel foods [19,20]. These variables are influenced by a complex interaction among sensory liking, perceived health benefits, familiarity, and cultural attitudes [21,22]. In particular, the relationship between liking and behavioural intention has been described as a central pathway linking sensory perception to real-world consumption behaviour [23,24]. However, evidence suggests that high sensory acceptance does not always translate into purchase or consumption, highlighting the need to integrate sensory and behavioural analyses [25].

Despite the growing scientific interest in baobab, most studies have primarily focused on its nutritional composition, functional properties, and technological applications [5,7], whereas consumer-oriented research remains relatively scarce. In particular, few studies have investigated the sensory drivers of consumer acceptance and consumer segmentation, especially in European populations, where familiarity with baobab remains limited. Furthermore, the relationships between sensory perception and behavioural outcomes, including intended consumption frequency and willingness to pay, have received little attention, hindering a comprehensive understanding of consumer acceptance of baobab fruit pulp [26]. A previous exploratory study conducted in Portugal reported moderate sensory acceptance and suggested promising market prospects; however, it was based on a relatively small sample and relied primarily on descriptive analyses [27]. Consequently, evidence regarding the sensory determinants of consumer acceptance and the behavioural factors influencing market adoption remain limited. The present study addresses these gaps by employing a larger consumer sample together with multivariate statistical approaches, including multiple regression and consumer segmentation analysis, to provide a more comprehensive understanding of baobab acceptance. Building upon these preliminary findings, the present study aimed to provide a more comprehensive understanding of the sensory and consumer acceptance of baobab fruit pulp in a Portuguese convenience sample. Specifically, it sought to (i) identify the sensory drivers of overall liking; (ii) segment consumers according to their sensory acceptance profiles; and (iii) examine whether consumer segments differed in behavioural outcomes, namely intended consumption frequency and willingness to pay.

## 2. Materials and Methods

### 2.1. Study Design, Participants, and Ethical Considerations

A cross-sectional consumer sensory study was conducted to evaluate sensory perception, overall liking, and behavioural responses towards baobab fruit pulp (*Adansonia digitata* L.), commonly referred to as múcua. The study protocol was approved by the Ethics Committee of the School of Health Sciences and Technologies, Universidade Lusófona (approval no. P2-26, 29 May 2023), and conducted in accordance with the Declaration of Helsinki [28]. Data collection and sensory evaluation were conducted between June and July 2023. Participants (≥18 years) were recruited through convenience sampling among adults living in Portugal. Although individuals with diverse sociodemographic backgrounds were included, the sample consisted predominantly of young adults and students, and therefore should not be considered representative of the Portuguese population. Consequently, the findings should be interpreted within the context of a Portuguese convenience sample, rather than being generalised to the wider population. Participants were informed about the objectives of the study and provided written informed consent before participation. Participants were screened to exclude individuals with known food allergies or intolerances to baobab or related products. All participants provided informed consent prior to participation. The study population consisted of 117 untrained consumers, reflecting real-market conditions, as recommended for consumer acceptance testing [14,15].

### 2.2. Sample Preparation and Presentation

Baobab pulp was manually separated from commercially available dried baobab fruit. The product was obtained from a single commercial batch and stored in its original sealed packaging in a cool, dry place, protected from direct light, until the day of sensory evaluation, following the manufacturer’s storage recommendations. The edible pulp was manually separated from seeds and fibrous material and subsequently homogenised using a hand blender (ErgoMixx Pro MS6CB6110, Bosch, BSH Hausgeräte GmbH, Munich, Germany) to ensure uniformity in texture and appearance. When necessary, sieving was performed to remove coarse particles and enhance consistency. Samples were prepared on the day of evaluation to preserve sensory integrity and minimise physicochemical changes that could influence perception [14]. All participants evaluated samples prepared from the same commercial batch to ensure consistency throughout the sensory evaluation. Each participant received approximately 30–50 g of baobab pulp, weighed using a digital precision balance (0.01 g readability; Relaxdays GmbH, Halle (Saale), Germany) and served at room temperature in identical, odourless, and coded containers to avoid visual bias [15].

### 2.3. Sensory Evaluation Conditions and Procedure

Sensory evaluation was conducted under controlled conditions designed to minimise external sources of variability, including environmental distractions and lighting effects—ISO 8589:2007 [29]. Participants were instructed to (i) visually assess the sample before tasting, (ii) place the sample in the mouth and allow adequate oral processing, and (iii) evaluate each attribute independently. Although only a single sample was evaluated, water was provided to standardise oral conditions and minimise residual effects. Data were collected using a structured, self-administered questionnaire accessed via QR code and completed on participants’ personal mobile devices.

### 2.4. Sensory Attributes and Measurement Scales

Participants evaluated the following sensory attributes: appearance, colour, aroma, texture, taste, sweetness, acidity, and overall liking. All sensory attributes were assessed using a 9-point hedonic scale (1 = dislike extremely; 9 = like extremely), a standard approach for measuring consumer acceptance and affective responses [14,30].

### 2.5. Behavioural Measures

In addition to sensory evaluation, behavioural responses were assessed to explore the relationship between perception and potential market acceptance. Intended consumption frequency was measured using a 9-point ordinal scale (0 = never; 8 = daily). Willingness to pay was assessed by asking participants to indicate the maximum price (€ per kg) they would be willing to pay, an approach commonly used to estimate perceived economic value and market potential [31].

### 2.6. Statistical Analysis

Statistical analyses were performed using IBM SPSS Statistics (version 26.0, IBM Corp., Armonk, NY, USA). Descriptive statistics were reported as medians and interquartile ranges (IQR) for ordinal variables, and as absolute and relative frequencies (*n*, %) for categorical variables, in accordance with recommendations for sensory and consumer data analysis [15]. To identify sensory drivers of overall liking, a multiple linear regression model was applied, with overall liking as the dependent variable and sensory attributes as independent predictors. Model performance was evaluated using the coefficient of determination (R^2^) and adjusted R^2^, while overall model significance was assessed using the F-test (ANOVA). Regression coefficients (β), standard errors, and associated *p*-values were reported. Consumer segmentation was performed using k-means cluster analysis based on sensory attribute ratings, a widely applied approach in consumer sensory science [32]. Cluster stability was assessed through convergence criteria and interpretability of cluster profiles. A two-cluster solution was retained to distinguish between lower-liking and higher-liking consumer segments. As cluster solutions are derived to maximise between-group differences, ANOVA results from the clustering procedure were interpreted descriptively. Differences between clusters were assessed using the Mann–Whitney U test, given the ordinal nature and non-normal distribution of sensory data [33]. Results were expressed as medians (IQR), U statistics, Z values, and *p*-values. Statistical significance was set at *p* < 0.05.

## 3. Results

### 3.1. Participant Characteristics

Participant characteristics are presented in Table 1. A total of 117 participants were included in the study. The median age was 23 years (IQR: 20–24). The sample was predominantly female (61.5%) and composed mainly of students (68.4%). Regarding nationality, 64.1% of participants were Portuguese, while 35.9% were from other nationalities. Most participants reported a willingness to try new foods (93.2%). A total of 66.7% indicated that it was their first time consuming baobab fruit pulp. The median monthly income was €575 (IQR: 0–1000), based on available data (*n* = 88).

### 3.2. Sensory Evaluation of Baobab Fruit Pulp

Sensory evaluation results are presented in Table 2. Overall liking showed a median score of 6 (IQR: 3–7), indicating moderate acceptance among participants. Taste, aroma, and acidity presented median values of 5, with relatively balanced distributions between disliking and liking responses. In contrast, sweetness had the lowest median score (3; IQR: 2–5) and the highest proportion of disliking responses (64.1%), indicating it as the least accepted attribute. Appearance and texture also showed lower median scores (median = 4), with a predominance of disliking responses, suggesting variability in visual and mouthfeel perception.

### 3.3. Sensory Evaluation According to Prior Exposure

Differences in sensory evaluation according to prior exposure to baobab fruit pulp are presented in Table 3. Participants who had previously consumed baobab fruit pulp reported significantly higher liking scores for appearance (*p* = 0.002), colour (*p* = 0.004), aroma (*p* = 0.001), texture (*p* = 0.001), and taste (*p* = 0.011) compared to first-time consumers. No significant differences were observed for sweetness (*p* = 0.260), acidity (*p* = 0.192), or overall liking (*p* = 0.062), although a trend towards higher overall liking was observed among participants with prior exposure.

### 3.4. Sensory Drivers of Overall Liking

The results of the regression analysis identifying the sensory drivers of overall liking are presented in Table 4. The model explained a high proportion of variance in overall liking (R^2^ = 0.823, *p* < 0.001), indicating a strong model fit. Taste emerged as the strongest predictor of overall liking (β = 0.362, *p* < 0.001), followed by texture (β = 0.182, *p* = 0.042) and acidity (β = 0.105, *p* = 0.045). These results suggest that flavour-related attributes play a central role in shaping consumer acceptance of baobab fruit pulp. In contrast, appearance, colour, aroma, and sweetness were not significant predictors of overall liking (*p* > 0.05), indicating that these attributes had a limited influence on consumer preference in this sample.

### 3.5. Intended Consumption Frequency and Willingness to Pay

Intended consumption frequency and willingness to pay are presented in Table 5. Overall, participants showed a moderate willingness to consume baobab fruit pulp, with the largest proportion reporting consumption once a month or less (22.2%), followed by 1–2 times per week (17.1%). A smaller proportion of participants indicated frequent consumption, including daily intake (6.8%) and 5–6 times per week (5.1%). The median willingness to pay was €3.0/kg (IQR: €2.0–€5.0), suggesting moderate price sensitivity among participants.

### 3.6. Consumer Segmentation

Consumer segmentation analysis identified two distinct clusters based on sensory evaluations of baobab fruit pulp (Table 6). Cluster 1 (*n* = 70) was characterised by low median scores across all sensory attributes, indicating a generally unfavourable perception of the product. In contrast, Cluster 2 (*n* = 47) showed substantially higher median ratings, reflecting a positive sensory evaluation profile.

Significant differences between clusters were observed for all evaluated attributes (*p* < 0.001). In particular, large differences were found for taste (median: 3 vs. 8), texture (3 vs. 8), and overall liking (4 vs. 8), highlighting these attributes as key differentiators between consumer segments.

Consumers in the high-liking cluster also reported significantly greater intended consumption frequency than those in the low-liking cluster (median: 4 vs. 1, *p* < 0.001).

Likewise, willingness to pay differed significantly between clusters, with participants in the high-liking cluster reporting a higher willingness to pay for baobab fruit pulp than those in the low-liking cluster (Mann–Whitney U = 1059.0, Z = −2.628, *p* = 0.009, r = 0.25).

Sociodemographic characteristics and previous exposure according to consumer clusters are presented in Table 7.

Previous exposure to baobab differed significantly between consumer clusters (χ^2^ = 5.177, *p* = 0.023), with previously exposed participants being more frequently represented in the high-liking cluster than first-time consumers. In contrast, sex was not associated with cluster membership (*p* = 0.905), while nationality showed only a non-significant trend (*p* = 0.077) (Table 7).

## 4. Discussion

**Figure 1:**
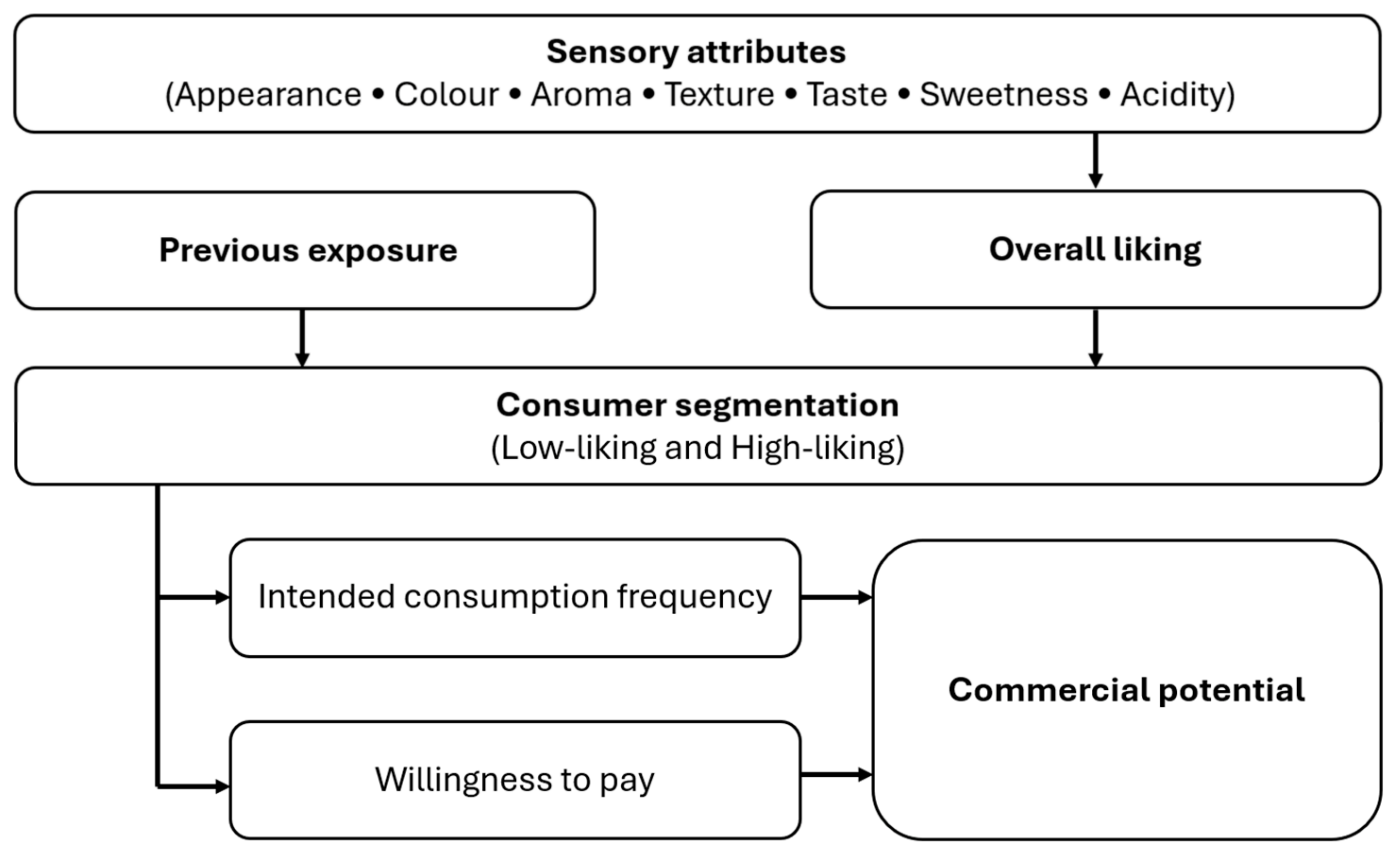
Conceptual synthesis of the associations observed between sensory perception, familiarity, consumer segmentation, intended consumption frequency, and willingness to pay in the present study. The framework summarises the principal relationships identified and is intended solely as a conceptual representation of the observed associations; it does not imply causal or predictive relationships.

This study provides new insights into the sensory perception, consumer segmentation, and behavioural responses associated with baobab pulp manually separated from commercially available dried baobab fruit in a Portuguese convenience sample. By combining hedonic evaluation with multivariate statistical analyses, the study extends previous consumer research by identifying the sensory attributes associated with overall liking, characterising distinct consumer segments, and exploring their associations with intended consumption frequency and willingness to pay. The conceptual framework presented in Figure 1 integrates these findings and summarises the principal associations observed between sensory perception and consumers’ behavioural responses.

### 4.1. Sensory Acceptance of Baobab Fruit Pulp

The moderate acceptance observed in the present study, together with the considerable inter-individual variability, indicates that baobab elicits heterogeneous consumer responses. This pattern is consistent with previous research showing that unfamiliar or culturally non-traditional foods often generate polarised hedonic evaluations owing to differences in familiarity, prior exposure, expectations, and food neophobia [34,35,36]. Although a previous Portuguese pilot study also reported generally favourable sensory acceptance of baobab pulp, its smaller sample size and descriptive analytical approach limited the identification of the factors underlying consumer acceptance [27]. By combining multivariate statistical analyses with consumer segmentation, the present study provides a more comprehensive understanding of the sensory and behavioural factors associated with baobab acceptance and highlights the value of segmentation approaches for identifying meaningful differences that may not be evident from average liking scores alone [32].

The regression analysis demonstrated that flavour-related attributes, particularly taste, followed by texture and acidity, were more strongly associated with overall liking than the remaining sensory characteristics. These findings are consistent with previous evidence showing that sensory quality, especially flavour perception, remains one of the principal determinants of acceptance for both conventional and novel foods, irrespective of their nutritional or functional properties [14,34,36,37]. Likewise, studies on functional and underutilised foods have consistently shown that flavour-related attributes exert a greater influence on consumer responses than perceived health benefits alone [14,34,36]. The prominent role of taste may reflect its function as an integrative sensory experience, combining gustatory, olfactory, and trigeminal stimuli into a single hedonic judgement [14,34,36].

As consumers ultimately evaluate foods through their overall flavour perception, rather than isolated sensory attributes, taste is expected to have a particularly important role during the first exposure to unfamiliar foods [37,38,39].

These findings emphasise the importance of flavour optimisation when developing baobab-based products intended for consumers with limited previous experience.

Texture was also positively associated with overall liking, reinforcing the importance of mouthfeel in consumer acceptance. Baobab pulp is naturally characterised by a dry, powdery texture and a slightly astringent mouthfeel, characteristics that may initially challenge consumers unfamiliar with the product. Similar barriers related to textural unfamiliarity have been described for other minimally processed and underutilised plant-based foods introduced into Western markets [13,40,41,42]. These observations suggest that the sensory challenges identified for baobab are unlikely to be unique, but instead reflect broader difficulties associated with introducing novel plant-based foods into culturally unfamiliar food environments.

Acidity also showed a modest but significant association with overall liking. Given the naturally high organic acid content of baobab pulp [3,7], achieving an appropriate balance between acidity and palatability may represent an important consideration during product development, as moderate acidity can enhance flavour perception, whereas excessive sourness may reduce consumer acceptance [14,43].

In contrast, appearance, colour, aroma, and sweetness were not significantly associated with overall liking. This may reflect the relatively limited variability of these attributes within the single-product design, together with the greater influence of flavour perception once tasting had occurred. Previous studies have similarly shown that visual and olfactory cues primarily shape consumers’ expectations before tasting, whereas flavour perception becomes the dominant determinant of hedonic evaluation during consumption [38,44].

### 4.2. Consumer Segmentation and Familiarity

The identification of two distinct consumer segments reinforces the heterogeneous nature of consumer responses towards baobab and highlights the value of segmentation approaches for evaluating novel foods. Rather than reflecting uniform consumer preferences, acceptance appears to vary considerably according to individual characteristics and previous experiences, a pattern consistently reported for other novel and functional foods [21,23,32,45]. These findings support the use of consumer segmentation as a complementary approach to average hedonic scores, enabling a more detailed understanding of the variability underlying consumer acceptance.

The behavioural differences observed between the two consumer segments further suggest that favourable sensory evaluations are associated with stronger behavioural responses, including greater intended consumption frequency and willingness to pay. This observation is consistent with theoretical frameworks proposing that hedonic evaluation plays an important role in shaping food choice, purchase intention, and consumers’ valuation of novel food products [21,23].

Familiarity also appears to contribute to consumer acceptance. Participants with previous experience of consuming baobab were more likely to belong to the high-liking segment, supporting the well-established familiarity effect whereby repeated exposure reduces uncertainty and food neophobia while increasing acceptance of unfamiliar foods [34,35,36,46]. A similar tendency was observed in our previous exploratory study, in which participants with prior experience of baobab generally reported more favourable hedonic evaluations [27]. Together, these findings suggest that familiarity may facilitate acceptance of baobab among consumers with limited previous exposure.

Nevertheless, previous exposure may also have influenced the observed associations between sensory attributes and overall liking, as familiar consumers may evaluate the product more positively than first-time consumers. Consequently, the relationship between familiarity and sensory acceptance should be interpreted with caution. Future studies using larger samples and multivariable analytical approaches are needed to determine the independent contribution of familiarity while accounting for potential confounding factors.

These findings also have implications for the introduction of baobab-based products into unfamiliar markets. Strategies that increase consumer familiarity, such as tasting opportunities, gradual product introduction, and educational initiatives highlighting appropriate culinary uses, may contribute to improving consumer acceptance. Similar approaches have been shown to facilitate acceptance of other novel and functional foods in European populations [13,16,19].

### 4.3. Consumer Behaviour and Implications for Product Development

The present findings suggest that favourable sensory evaluations are associated with more positive behavioural responses towards baobab, particularly greater intended consumption frequency and willingness to pay. These observations are consistent with previous studies showing that hedonic evaluations play an important role in shaping consumer responses to novel foods by influencing both intended consumption frequency and perceived product value [25,36].

Despite these positive associations, the overall moderate intended consumption frequency observed in the present study indicates that sensory acceptance alone is unlikely to ensure regular consumption. Consumer behaviour towards novel foods is widely recognised as a multidimensional process influenced not only by sensory perception, but also by familiarity, perceived health benefits, trust, food neophobia, cultural acceptance, and previous consumption experiences [35,46]. Consequently, improving sensory quality represents only one component of a broader strategy to promote consumer acceptance.

The association between higher sensory acceptance and greater willingness to pay further suggests that favourable sensory experiences contribute to consumers’ perceived value of baobab. Similar relationships between sensory acceptance, purchase intention, and willingness to pay have been reported for functional and innovative foods, reinforcing the importance of sensory quality in consumers’ evaluation of novel food products [16,19,21,34]. From a product development perspective, these findings indicate that optimising flavour and texture may improve consumer acceptance and support more favourable behavioural responses.

Because willingness to pay was assessed using a hypothetical purchasing scenario, these findings should be interpreted with caution. Although the observed differences between consumer segments provide useful insights into perceived product value, future studies using experimental auction methods, discrete choice experiments, or real purchasing scenarios are needed to determine consumers’ actual willingness to pay under market conditions.

Collectively, these findings suggest that the successful introduction of baobab into European food markets will depend on more than communicating its nutritional and functional properties. Consumer acceptance is also likely to be influenced by sensory quality, familiarity with the product, and perceived value. Strategies combining sensory optimisation with consumer education, tasting opportunities, and gradual exposure may therefore facilitate familiarity and contribute to greater acceptance among consumers with limited previous experience of baobab. These relationships are summarised in the conceptual framework presented in Figure 1, which provides an integrated overview of the principal associations identified in the present study without implying causal or predictive pathways.

## 5. Strengths and Limitations

This study presents several methodological strengths. Compared with previous research, it employed a larger consumer sample (*n* = 117) and a standardised 9-point hedonic scale, consistent with recommendations for consumer sensory testing [14]. The combination of validated sensory measurements with multivariate statistical techniques, including multiple regression and cluster analysis, enabled both the identification of the sensory drivers of overall liking and the characterisation of distinct consumer segments [32]. Furthermore, integrating sensory evaluation with behavioural outcomes, namely intended consumption frequency and willingness to pay, provided a more comprehensive assessment of consumer acceptance and behavioural responses. Building upon our previous Portuguese pilot study [27], the present work substantially extends the available evidence by offering a more robust and detailed understanding of the sensory and consumer acceptance of baobab fruit pulp.

Nevertheless, several limitations should be acknowledged. The use of convenience sampling, with a sample composed predominantly of young adults and students, limits the generalisability of the findings beyond the study population. Furthermore, approximately one-third of participants were of non-Portuguese nationality; therefore, the findings should not be considered representative of the Portuguese population. In addition, the relatively low median monthly income and the proportion of missing income data may have influenced participants’ stated willingness to pay.

The evaluation of a single product sample restricted the ability to explore optimal sensory formulations or perform preference mapping analyses, which are commonly recommended in sensory optimisation studies [47,48]. The cross-sectional design also prevented assessment of changes in liking following repeated exposure, an important determinant of novel food acceptance [35]. Moreover, psychographic variables such as food neophobia, health perceptions, and previous familiarity were not formally assessed, although they are recognised determinants of consumer acceptance of novel and functional foods [34,36].

Finally, willingness to pay was assessed using a hypothetical stated preference approach rather than an experimental economic method. Consequently, the reported values should be interpreted as indicative behavioural intentions rather than actual purchasing behaviour under real market conditions.

Future research should include larger and more diverse consumer samples, evaluate multiple baobab formulations, and apply advanced sensory methodologies such as preference mapping, temporal sensory analysis, and experimental purchasing designs. Longitudinal studies incorporating repeated exposure, psychographic variables, and discrete choice experiments would provide a more comprehensive understanding of consumer acceptance and behavioural responses towards baobab and other underutilised foods.

## 6. Conclusions

This study provides novel insights into the sensory perception, consumer segmentation, and behavioural responses associated with baobab pulp manually separated from commercially available dried baobab fruit in a Portuguese convenience sample. Taste emerged as the principal predictor of overall liking, followed by texture and acidity, highlighting the importance of flavour-related attributes in consumer acceptance. Two distinct consumer segments were identified, reflecting substantial heterogeneity in sensory preferences and demonstrating the usefulness of consumer segmentation for understanding the acceptance of novel foods.

Previous exposure to baobab was associated with greater sensory acceptance, suggesting that familiarity may facilitate the acceptance of underutilised foods. Furthermore, consumers belonging to the high-liking segment reported significantly greater intended consumption frequency and willingness to pay, indicating higher perceived product value and more favourable behavioural responses within the study sample.

Future research should evaluate different product formulations, include larger and more representative consumer populations, and investigate the influence of psychological and cultural determinants, such as food neophobia, health perceptions, and familiarity, on long-term acceptance and purchasing behaviour. Although these findings contribute to the understanding of consumer acceptance of baobab fruit pulp, broader conclusions regarding consumer behaviour and market acceptance in Portugal should await confirmation in representative population samples.

## Figures and Tables

**Table 1:** Sociodemographic characteristics of the participants (*n* = 117).

Characteristics	Median (IQR)	Characteristics	*n* (%)
Age (years)	23 (20–24)	Occupation	
Monthly income (€)	575 (0–1000) *	Student	80 (68.4)
		Working student	29 (24.8)
Characteristics	*n* (%)	Other	8 (6.8)
Sex		Willingness to try new foods	
Female	72 (61.5)	Yes	109 (93.2)
Male	45 (38.5)	No	8 (6.8)
Nationality		First time consuming baobab	
Portuguese	75 (64.1)	Yes	78 (66.7)
Other	42 (35.9)	No	39 (33.3)

* Income data available for *n* = 88 (24.8% missing).

**Table 2:** Sensory evaluation of baobab fruit pulp (*n* = 117).

Attribute	Median (IQR)	Disliking (%)	Neutral (%)	Liking (%)
Appearance	4 (3–7)	56.4	12.8	30.8
Colour	5 (3–8)	47.9	16.2	35.9
Aroma	5 (3–7)	47.9	15.4	36.8
Texture	4 (3–7)	51.3	8.5	40.2
Taste	5 (3–7)	47.9	10.3	41.9
Sweetness	3 (2–5)	64.1	12.0	23.9
Acidity	5 (3–7)	47.0	9.4	43.6
Overall liking	6 (3–7)	39.3	10.3	50.4

Data are presented as median (interquartile range). The 9-point hedonic scale ranged from 1 (“disliked extremely”) to 9 (“liked extremely”). Disliking corresponds to scores 1–4, neutral to score 5, and liking to scores 6–9. All variables were treated as ordinal.

**Table 3:** Sensory evaluation according to prior exposure to baobab fruit pulp.

Attribute	First-Time Consumers (*n* = 78) Median (IQR)	Previous Consumers (*n* = 39) Median (IQR)	*p*-Value
Appearance	4 (2.75–5)	5 (4–9)	0.002
Colour	4 (3–6.25)	7 (4–9)	0.004
Aroma	4 (3–6)	6 (4–9)	0.001
Texture	4 (3–6)	7 (4–9)	0.001
Taste	5 (3–7)	5 (4–9)	0.011
Sweetness	3 (2–5)	3 (2–7)	0.260
Acidity	5 (3–7)	4 (3–7)	0.192
Overall liking	5 (3–7)	6 (4–9)	0.062

Data are presented as median (interquartile range). Differences between groups were assessed using the Mann–Whitney U test. The 9-point hedonic scale ranged from 1 (“disliked extremely”) to 9 (“liked extremely”).

**Table 4:** Sensory drivers of overall liking of baobab fruit pulp.

Variable	B	SE	β	*p*-Value
Appearance	0.070	0.095	0.070	0.461
Colour	0.109	0.094	0.113	0.247
Aroma	0.161	0.097	0.152	0.101
Texture	0.183	0.089	0.182	0.042 *
Taste	0.340	0.080	0.362	<0.001 **
Sweetness	0.035	0.058	0.035	0.547
Acidity	0.107	0.053	0.105	0.045 *

Linear regression model identifying sensory drivers of overall liking. * *p* < 0.05; ** *p* < 0.001.

**Table 5:** Intended consumption frequency and willingness to pay for baobab fruit pulp.

Variable	Category	*n* (%)
Intended consumption frequency (*n* = 117)		
	Never	16 (13.7)
	Once a month or less	26 (22.2)
	Fortnightly	10 (8.5)
	3–4 times per month	18 (15.4)
	1–2 times per week	20 (17.1)
	3–4 times per week	13 (11.1)
	5–6 times per week	6 (5.1)
	Daily	8 (6.8)
Willingness to pay (€/kg) (*n* = 112)		
	Median	3.0
	P25–P75	2.0–5.0

**Table 6:** Comparison of sensory responses, intended consumption frequency and willingness to pay between the low- and high-liking consumer clusters.

Attribute	Cluster 1: Low-Liking Consumers (*n* = 70)	Cluster 2: High-Liking Consumers (*n* = 47)			
	Median (IQR)	Median (IQR)	U	Z	*p*-Value
Appearance	3 (2–4)	7 (5–9)	95.5	−8.679	<0.001
Colour	4 (3–4)	8 (7–9)	120.5	−8.547	<0.001
Aroma	4 (3–4)	7 (6–9)	133.5	−8.456	<0.001
Texture	3 (3–4)	8 (6–9)	108.5	−8.601	<0.001
Taste	3 (2.75–4)	8 (7–9)	109.0	−8.581	<0.001
Sweetness	3 (1–4)	6 (3–7)	657.5	−5.500	<0.001
Acidity	4 (3–5)	7 (6–8)	602.0	−5.803	<0.001
Overall liking	4 (3–5)	8 (7–9)	101.0	−8.624	<0.001
Intended consumption frequency	1 (1–3)	4 (3–5)	749.5	−4.992	<0.001
Willingness to pay (€ kg^−1^)	2.75 (1.63–4.38)	3.00 (2.13–5.75)	1059.0	−2.628	0.009

Notes: Data are presented as median (interquartile range, IQR). Differences between clusters were assessed using the Mann–Whitney U test. Sensory attributes and overall liking were assessed using a 9-point hedonic scale (1 = dislike extremely; 9 = like extremely). Intended consumption frequency was assessed using an 8-point ordinal scale ranging from “never” to “daily”.

**Table 7:** Sociodemographic characteristics according to consumer clusters.

Variable	Low-Liking *n* (%)	High-Liking *n* (%)	χ^2^	*p*
Female	44 (61.1)	28 (38.9)	0.014	0.905
Male	27 (60.0)	18 (40.0)		
Portuguese	50 (66.7)	25 (33.3)	3.135	0.077
Other nationalities	21 (50.0)	21 (50.0)		
First exposure	53 (67.9)	25 (32.1)	5.177	0.023
Previous exposure	18 (46.2)	21 (53.8)		

## Data Availability

The original contributions presented in this study are included in the article. Further inquiries can be directed to the corresponding authors.

## References

[1] Knez M., Ranić M., Gurinović M. (2024). Underutilized plants increase biodiversity, improve food and nutrition security, reduce malnutrition, and enhance human health and well-being. Let’s put them back on the plate!. Nutr. Rev..

[2] Kaur S., Kaur G., Kumari A., Ghosh A., Singh G., Bhardwaj R., Kumar A., Riar A. (2025). Resurrecting forgotten crops: Food-based products from potential underutilized crops a path to nutritional security and diversity. Future Foods.

[3] Chadare F.J., Linnemann A.R., Hounhouigan J.D., Nout M.J., Van Boekel M.A. (2009). Baobab food products: A review on their composition and nutritional value. Crit. Rev. Food Sci. Nutr..

[4] Kamatou G.P.P., Vermaak I., Viljoen A.M. (2011). An updated review of Adansonia digitata: A commercially important African tree. S. Afr. J. Bot..

[5] Braca A., Sinisgalli C., De Leo M., Muscatello B., Cioni P.L., Milella L., Ostuni A., Giani S., Sanogo R. (2018). Phytochemical Profile, Antioxidant and Antidiabetic Activities of *Adansonia digitata* L. (Baobab) from Mali, as a Source of Health-Promoting Compounds. Molecules.

[6] Silva M.L., Rita K., Bernardo M.A., Mesquita M.F.D., Pintão A.M., Moncada M. (2023). *Adansonia digitata* L. (Baobab) Bioactive Compounds, Biological Activities, and the Potential Effect on Glycemia: A Narrative Review. Nutrients.

[7] Tembo D.T., Holmes M.J., Marshall L.J. (2017). Effect of thermal treatment and storage on bioactive compounds, organic acids and antioxidant activity of baobab fruit (*Adansonia digitata*) pulp from Malawi. J. Food Compos. Anal..

[8] Abdulwaliyu I., Arekemase S., Batari M., Oshodin J., Mustapha R., Ibrahim D., Ekere A., Olusina O. (2024). Nutritional and pharmacological attributes of baobab fruit pulp. Food Prod. Process. Nutr..

[9] Buchmann C., Prehsler S., Hartl A., Vogl C.R. (2010). The Importance of Baobab (*Adansonia digitata* L.) in Rural West African Subsistence—Suggestion of a Cautionary Approach to International Market Export of Baobab Fruits. Ecol. Food Nutr..

[10] Offiah V.O., Falade K.O. (2023). Potentials of baobab in food systems. Appl. Food Res..

[11] Gebauer J., El-Siddig K., Ebert G. (2002). Baobab (*Adansonia digitata* L.): A review on a multipurpose tree with promising future in the Sudan. Eur. J. Hortic. Sci..

[12] European Commission (2008). Commission decision of 27 June 2008 authorising the placing on the market of baobab dried fruit pulp as a novel food ingredient under Regulation (EC) No 258/97 of the European Parliament and of the Council. Off. J. Eur. Union.

[13] Günden C., Atakan P., Yercan M., Mattas K., Knez M. (2024). Consumer Response to Novel Foods: A Review of Behavioral Barriers and Drivers. Foods.

[14] Lawless H.T., Heymann H. (2010). Sensory Evaluation of Food: Principles and Practices.

[15] Meilgaard M.C., Civille G.V., Carr B.T. (2015). Sensory Evaluation Techniques.

[16] Lu P., Parrella J.A., Xu Z., Kogut A. (2024). A scoping review of the literature examining consumer acceptance of upcycled foods. Food Qual. Prefer..

[17] Jürkenbeck K., von Steimker F., Spiller A. (2024). Consumer’s perception of food pairing products with usual, novel and unusual flavour combinations: A segmentation approach. Appetite.

[18] Cunha L.M., Lima R.C., Ribeiro J.C., Rocha C., Costa A.I.D.A., Monteiro M.J.P., Lamy E. (2024). Rapid Sensory Profiling Methods for Research and Industrial Applications. Sensory Evaluation and Consumer Acceptance of New Food Products: Principles and Applications.

[19] Baker M.T., Lu P., Parrella J.A., Leggette H.R. (2022). Consumer Acceptance toward Functional Foods: A Scoping Review. Int. J. Environ. Res. Public Health.

[20] Marin A., Rodino S., Pop R.-E., Dragomir V., Butu M. (2025). Consumer Attitudes, Awareness, and Purchase Behaviour for Certified Mountain Products in Romania. Sustainability.

[21] Grunert K.G., Hieke S., Wills J. (2014). Sustainability Labels on Food Products: Consumer Motivation, Understanding and Use. Food Policy.

[22] Głuchowski A., Czarniecka-Skubina E., Kostyra E., Wasiak-Zys G., Bylinka K. (2021). Sensory Features, Liking and Emotions of Consumers towards Classical, Molecular and Note by Note Foods. Foods.

[23] Köster E.P. (2009). Diversity in the determinants of food choice: A psychological perspective. Food Qual. Prefer..

[24] Hallikainen H., Halinen M., Tervonen H., Ruusunen N., Wang Y. (2025). Rating with the senses: How sensory encounters are reflected on online review ratings?. Psychol. Mark..

[25] Mustapa M.A.C., Kallas Z., Silande C., Gagnaire V., Jan G., López-Mas L., Aguiló-Aguayo I. (2024). From taste to purchase: Understanding the influence of sensory perceptions and informed tasting on plant-based product purchases—An extension of the theory of planned behavior. J. Agric. Food Res..

[26] Sagha M., Seyyedamiri N., Foroudi P., Akbari Sadr M. (2022). The One Thing You Need to Change Is Emotions: The Effect of Multi-Sensory Marketing on Consumer Behavior. Sustainability.

[27] Tomás A., Oliveira L. (2023). Mucua: Sensory analysis of the fruit of *Adansonia digitata* L. (Baobab) in natura. Biomed. Biopharm. Res..

[28] World Medical Association (2013). Declaration of Helsinki: Ethical principles for medical research involving human subjects. JAMA.

[29] (2007). Sensory Analysis-General Guidance for Design of Test Rooms.

[30] Teixeira L.V. (2013). Análise sensorial na indústria de alimentos. Rev. Inst. Latic. Cândido Tostes.

[31] Lusk J.L., Shogren J.F. (2007). Experimental Auctions: Methods and Applications in Economic and Marketing Research.

[32] Varela P., Ares G. (2014). Novel Techniques in Sensory Characterization and Consumer Profiling.

[33] Field A. (2024). Discovering Statistics Using IBM SPSS Statistics.

[34] Siegrist M. (2008). Factors influencing public acceptance of innovative food technologies and products. Trends Food Sci. Technol..

[35] Tuorila H., Hartmann C. (2020). Consumer responses to novel and unfamiliar foods. Curr. Opin. Food Sci..

[36] Verbeke W. (2006). Functional foods: Consumer willingness to compromise on taste for health?. Food Qual. Prefer..

[37] Delarue J., Lawlor B., Rogeaux M. (2015). Rapid Sensory Profiling Techniques. Applications in New Product Development and Consumer Research.

[38] Spence C., Okajima K., Cheok A.D., Petit O., Michel C. (2016). Eating with our eyes: From visual hunger to digital satiation. Brain Cogn..

[39] Wolinska-Kennard K., Schönberger C., Fenton A., Sahin A.W. (2025). Mouthfeel of Food and Beverages: A Comprehensive Review of Physiology, Biochemistry, and Key Sensory Compounds. Compr. Rev. Food Sci. Food Saf..

[40] Szczesniak A.S. (2002). Texture is a sensory property. Food Qual. Prefer..

[41] Muhammad A.I., Rilwan A., Nouruddeen Z.B., Ejiohuo O., Al-Habsi N. (2025). Enhancing the Sensory Quality, Stability, and Shelf Life of Baobab Fruit Pulp Drinks: The Role of Hydrocolloids. Polymers.

[42] Ares G., Varela P. (2018). Methods in consumer research. New Approaches to Classic Methods.

[43] Köster E.P., Mojet J. (2015). From mood to food and from food to mood: A psychological perspective on the measurement of food-related emotions in consumer research. Food Res. Int..

[44] Biswas D., Labrecque L.I., Lehmann D.R. (2021). Effects of Sequential Sensory Cues on Food Taste Perception: Cross-Modal Interplay Between Visual and Olfactory Stimuli. J. Consum. Psychol..

[45] Dolnicar S. (2002). A Review of Unquestioned Standards in Using Cluster Analysis for Data-Driven Market Segmentation. Proceedings of the Faculty of Commerce—Papers. https://ro.uow.edu.au/articles/conference_contribution/A_Review_of_Unquestioned_Standards_in_Using_Cluster_Analysis_for_Data-Driven_Market_Segmentation/27692220?file=50430537.

[46] Siegrist M., Hartmann C. (2020). Consumer acceptance of novel food technologies. Nat. Food.

[47] Ares G. (2018). Methodological issues in cross-cultural sensory and consumer research. Food Qual. Prefer..

[48] Ruiz-Capillas C., Herrero A.M. (2021). Sensory Analysis and Consumer Research in New Product Development. Foods.

